# The Hospital World

**Published:** 1911-05-06

**Authors:** 


### The Hospital World.

### A Useful Memorial to a Committeeman.

It is interesting to learn that an attempt is being made by the friends of the late Mr. John Howard Colls to endow a cot in memory of his seventeen years' service as committeeman of the Evelina Hospital for Sick Children. So much quiet work is done for hospitals that it is always gratifying to record an appreciation that will carry on the work of the man in whose honour it is given.

### Tribute to a Hospital Secretary.

The successful progress of the extension scheme at the North Ormesby Hospital, Middlesbrough, the striking support which the Middlesbrough employees have given to their hospital, and the success of certain administrative changes have led the Hospital's Council to make the following personal tribute : "They desire to place on record their great appreciation of the excellent services rendered by the Secretary, Mr. E. C. Smith, in regard to the carrying out of the structural alterations in the hospital, and also to the introduction of many beneficial alterations in the administration of the affairs of the institution, whereby, owing to the efficient manner in which these have been carried out by the Mother and Sisters, the cost per bed has been very considerably reduced."—An account of these developments will be found on page 148.

### The "Busk" Memorial at the Dreadnought Hospital.

On Thursday last week Mr. Perceval Alleyn Nairne, Chairman of the Seamen's Hospital Society, unveiled at the Dreadnought Hospital a memorial tablet to the late Mr. George Busk, F.R.S., F.R.C.S., one of the most distinguished surgeons of his day. This tablet is erected over the bed which has been endowed by the Misses Elinor and Frances Busk in memory of their father. Mr. Busk served for no less than fifty-six years on the staff of the Seamen's Hospital Society, having been appointed "apothecary" in 1830, when the work of the Society was carried on on board the *Grampus*, a fifty-gun vessel lying in the River Thames. In 1832 he was appointed assistant surgeon, and in 1839 surgeon. In 1854 he became visiting surgeon, and in 1866 consulting surgeon. He died in 1886. Mr. Busk was Hunterian Professor to the Royal College of Surgeons from 1856 to 1859, an office afterwards held by Huxley. In 1863 he was elected to the Council of the College, and retained his seat until 1880, when he retired. He served on the Board of Examiners in 1868, was elected Vice-President for 1869 and 1870, and President in 1871. He took the keenest interest in the museum of the College, which owes to him many valuable specimens, including a skull which represents one of the earliest known forms of man. In addition, Mr. Busk laid the foundations of a perfect collection of skulls, which is conserved in the museum of the hospital at Greenwich. This collection contains many objects which are absolutely unique. There hangs in the Physicians' Room at the Dreadnought Hospital a very fine engraving of the granting of the Charter to the Royal College of Surgeons by Henry VIII., which was a gift from Mr. Busk.

At the ceremony on Thursday last there assembled a small but interesting gathering of men and women, most of whom had had association with Mr. Busk, and some of whom had been closely connected with him in his working life. After the Chairman had unveiled the tablet and expressed the thanks of the Seamen's Hospital Society for this gift, Mr. G. H. Makins, C.B., surgeon of St. Thomas's Hospital, spoke of his association with Mr. Busk, and laid stress upon the deep indebtedness of the medical profession of to-day to his great ability. The tablet, which is of white marble, bears the following inscription :—

"In memory of George Busk, F.R.S., President of the Royal College of Surgeons, 1871, whose skill and unwearying devotion were ever at the service of the Seamen's Hospital Society from 1830 to 1886. This bed is endowed by his daughters in fulfilment of the wish of their mother, Ellen Busk."

### The Taunton Hospital and the Portman Family.

The Hon. Edward William Berkeley Portman, eldest son of Viscount Portman, who died on Thursday last week, followed the family tradition, and was a friend most valued by the Taunton and Somerset Hospital, not merely for his munificent contributions, but even more for the intense interest and business energy which, as President and Chairman of the Committee of Governors, he displayed in its general management.

The Portman family has been for centuries intimately connected with West Somerset, and owns large landed estates in the vicinity of Taunton. For many years prior to his death in 1888, the first Viscount was patron of the hospital, and in that year the Hon. W. H. B. Portman, the present Viscount, held the office of president, and subsequently accepted the position of patron, which he still occupies. During his presidency, the Queen Victoria Jubilee Nursing Institute and the new out-patient department, erected in commemoration of the Jubilee, were opened.

Mr. Edward Portman, after coming to live at Hestercombe, near Taunton, very soon began to take an active interest in the institution, and in the year 1897 he was elected its president. His keenness was brought about in this way. There came from Bristol to reside near Taunton the late Mr. Proctor Baker, who had for long been president of the Bristol General Hospital. He soon brought his ripe experience of hospital management to bear upon the Somerset institution, and during 1901 assisted Mr. Portman in promoting a scheme for the entire remodelling of the older portions of the building. Mr. Portman succeeded to the presidency in 1904, and has ever since held the office, and it was almost entirely owing to the initiative, the liberality, and the leadership of these two gentlemen that the scheme was successfully completed within six years at the cost of nearly £12,000. The scheme included, amongst many minor improvements, better accommodation for the servants and more rooms and a larger dining-hall for the nursing staff, the removal of the kitchen to the top storey of the house, a hot-water heating system, a re-constructed theatre, and covered balconies for the main wards. It is interesting to remember that the County Hospital, originally built in commemoration of the Jubilee of the reign of King George III., was opened to patients on March 25, 1812, and Mr. Portman had already began to plan proposals for celebrating its centenary in March, 1912. At the last annual meeting of the Governors he foreshadowed as a part of those proposals the raising of an endowment fund of £30,000, to which, it is understood, his personal contribution was to be £5,000, and he had also enlisted the sympathy of many leading men in the county. Mr. Portman possessed means, persuasive influence, and business talent, all of which he employed ungrudgingly for the benefit of the County Hospital.

